# Connectivity, persistence, and loss of high abundance areas of a recovering marine fish population in the Northwest Atlantic Ocean

**DOI:** 10.1002/ece3.3495

**Published:** 2017-10-18

**Authors:** Stephanie A. Boudreau, Nancy L. Shackell, Stuart Carson, Cornelia E. den Heyer

**Affiliations:** ^1^ Gulf Fisheries Centre Fisheries and Oceans Canada Moncton NB Canada; ^2^ Bedford Institute of Oceanography Fisheries and Oceans Canada Dartmouth NS Canada; ^3^ Department of Mathematics and Statistics Dalhousie University Halifax NS Canada

**Keywords:** Atlantic halibut, commercial fisheries, fisheries recruitment, habitat protection, Northwest Atlantic Ocean, persistent areas of abundance, R‐INLA, spatiotemporal analysis

## Abstract

In the early 1990s, the Northwest Atlantic Ocean underwent a fisheries‐driven ecosystem shift. Today, the iconic cod (*Gadus morhua*) remains at low levels, while Atlantic halibut (*Hippoglossus hippoglossus*) has been increasing since the mid‐2000s, concomitant with increasing interest from the fishing industry. Currently, our knowledge about halibut ecology is limited, and the lack of recovery in other collapsed groundfish populations has highlighted the danger of overfishing local concentrations. Here, we apply a Bayesian hierarchical spatiotemporal approach to model the spatial structure of juvenile Atlantic halibut over 36 years and three fisheries management regimes using three model parameters to characterize the resulting spatiotemporal abundance structure: persistence (similarity of spatial structure over time), connectivity (coherence of temporal pattern over space), and spatial variance (variation across the seascape). Two areas of high juvenile abundance persisted through three decades whereas two in the northeast are now diminished, despite the increased abundance and landings throughout the management units. The persistent areas overlap with full and seasonal area closures, which may act as refuges from fishing. Connectivity was estimated to be 250 km, an order of magnitude less than the distance assumed by the definition of the Canadian management units (~2,000 km). The underlying question of whether there are distinct populations within the southern stock unit cannot be answered with this model, but the smaller ~250 km scale of coherent temporal patterns suggests more complex population structure than previously thought, which should be taken into consideration by fishery management.

## 1 INTRODUCTION

Atlantic halibut (*Hippoglossus hippoglossus*; halibut), a large semi‐pelagic flatfish, has a broad range, spanning several ecosystems and international boundaries. The range of halibut extends from the United States of America's (USA) Atlantic region of southern New England, across the international maritime border throughout Atlantic Canada to the Canadian Arctic. The range also includes regions outside of the exclusive economic zone (EEZ), such as the French territory of St. Pierre and Miquelon, Greenland, and Iceland (Froese & Pauly, 2015; Trumble, Neilson, Bowering, & McCaughran, 1993). Presently, the Canadian Atlantic halibut commercial fishery is managed as two discrete stocks, the larger of the two is referred to as the Scotian Shelf‐southern Grand Banks management unit (DFO, 2015a), and encompasses the waters from the Gulf of Maine off of southwest Nova Scotia to southeastern Newfoundland and Labrador. The smaller stock is contained in the Gulf of St. Lawrence (DFO, 2015b).

**Figure 1:**
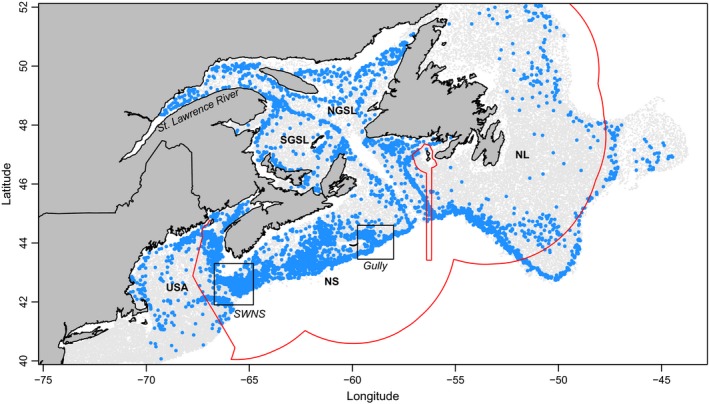
Juvenile halibut presence (raw data from ecosystem trawl surveys) indicated in blue, absence in gray, and the exclusive economic zone (EEZ) in red. St. Pierre and Miquelon French territory is denoted by inset EEZ. Regions of analysis are marked; Newfoundland and Labrador (NL), Nova Scotia (the Scotian Shelf and Gulf of Maine; NS), Northern Gulf of St. Lawrence (NGSL), Southern Gulf of St. Lawrence (SGSL), and the United States of America (the survey set from NMFS includes tows into Canadian waters; USA). Areas of interest for juvenile halibut abundance are also marked: the Gully, Southwest Nova Scotia (SWNS), and are denoted generally with a box. The St. Lawrence River estuary is also identified, and while there is halibut present; ultimately, it was excluded from the formal analysis (see Methods)

The Canadian Atlantic halibut fishery was largely unregulated until 1988, when the management units were established, and a minimum legal length for commercial harvest was introduced to maximize sustainable yield (Neilson & Bowering, 1989; Trumble et al., 1993). Although admittedly data limited, the definition of “one large stock” for the Scotian Shelf‐southern Grand Banks management unit was based primarily on tagging results which indicated the intermixing of Newfoundland and Scotian Shelf halibut, but not those in the Gulf of St. Lawrence Neilson, Bowering, & Frechet, 1987; Figure 1) and supported partially by a recent study on electronically tagged halibut in the Gulf of St. Lawrence (Le Bris et al., 2017). Additionally, length at age comparisons (Bowering, 1986) suggested differences in demographic rates.

There is a long history of halibut exploitation on the Atlantic coast. Caught first as bycatch in the Atlantic cod fisheries, there are records of abundant halibut in the near shore of Massachusetts Bay during the early 1800s (Grasso, 2008). The directed fishery began there in the 1830s, but sequentially moved further offshore to maintain catch rates. Effort then shifted northward throughout Canadian waters, again moving sequentially as stocks were depleted, eventually exploiting populations as far north as the Davis Strait of the Canadian Arctic in 1866 (Grasso, 2008; Trumble et al., 1993). The halibut fishery continued in Canadian waters throughout the early 1900s, with core fishing areas in southwest Nova Scotia, along the continental shelf edge south of Newfoundland and near the Gully, a deepwater canyon on the edge of the Scotian Shelf (now a marine protected area), and in the Gulf of St. Lawrence (McCracken, 1958; Figure 1). Decades of fishing pressure culminated in large declines of halibut and the fishery through the 1990s (Trzcinski & Bowen, 2016).

Halibut landings in Atlantic Canada have been steadily increasing since the early 2000s (DFO, 2015a, 2015b; Trzcinski & Bowen, 2016) where the Scotian Shelf‐southern Grand Banks Atlantic halibut management unit was certified by the Marine Stewardship Council in 2013 (Martell, Vincent, & Turis, 2013). This stands in stark contrast to the status of halibut at the southern end of the species’ range in the United States where the commercial halibut fishery has been under moratorium since 1999 (Department of Commerce (DC), 1999). Additionally, the United States included the Canadian waters when halibut was designated a “Species of Concern” under the USA *Endangered Species Act* in 2004 (Northeast Fisheries Science Center (NEFSC), 2012). Juvenile halibut abundance in Canada from 1965 to 2013 was estimated to be approximately fivefold greater than in the United States despite ample suitable habitat in US waters (Shackell, Frank, Nye, & den Heyer, 2016). This suggests that halibut dynamics occur on a much smaller scale than currently assumed and that US halibut have never fully recovered from historical overfishing (Shackell et al., 2016). Recent (den Heyer et al., 2012; Kanwit, 2007; Seitz, Evans, Courtney, & Kanwit, 2016; Seitz, Farrugia, Norcross, Loher, & Nielsen, 2017) and historical tagging analyses (McCracken, 1958) provide support for the hypothesis that halibut may exist as a series of local populations.

The historical fishing pattern of American fleets on halibut suggests serial local overfishing (Maury & Gascuel, 2001), that is, sequentially exploiting subpopulations to maintain catch levels (Grasso, 2008). Fishing subpopulations can lead to local extinction by exerting strong fishing pressure and potentially collapsing small weakly connected subpopulations with slower growth and recruitment rates (Frank & Brickman, 2000; Kerr, Cadrin, & Secor, 2010; Reich & Dealteris, 2009; Sterner, 2007). Examples of local overfishing in temperate waters (reviewed in Ciannelli et al., 2013 and Safina, Rosenberg, Myers, Quinn, & Collie, 2005) include North Sea herring (*Clupea harrengus*; Payne, 2010; Ruzzante et al., 2006) and the Northwest (NW) Atlantic's northern cod (Hutchings, 1996; Rose, Kulka, Goddard, & Fletcher, 2000). The northern cod was aggressively fished by fishermen seeking high‐density areas of cod (Hutchings, 1996), ultimately targeting discrete spawning areas, which lead to the disappearance of subpopulations and the spatial erosion of the northern cod metapopulation (Hu & Wroblewski, 2009; Smedbol & Wroblewski, 2002). In fact, cod, across its NW Atlantic range, has not recovered from a basin‐wide collapse in the early 1990s resulting from decades of overfishing (Shelton, Sinclair, Chouinard, Mohn, & Duplisea, 2006). It is now understood that the spatial scale of fisheries management was much larger than that of the subpopulations (i.e., Roney et al., 2016).

The importance of understanding population spatial structure was acknowledged by fisheries scientists during the late 1800s; however, since the 1960s, fisheries science has focused on the development of quantitative methods to assess the amount of fishable biomass (Stephenson, 2002), with an underlying assumption that any locally depleted population would be replenished by neighboring areas (e.g., Svedäng, Cardinale, & André, 2001). While it is possible for recolonization to occur (Corten, 2013), when there is a lack of recovery in local areas, it suggests that recolonization from elsewhere is not a simple density‐dependent response and may be attributed to philopatric behavior (Svedäng et al., 2001; Svedäng & Svenson, 2006) or changes in population structure or demographic rates (Payne, 2010), or that recolonization rates cannot counter excessive fishing pressure (e.g., Shackell, Frank, & Brickman, 2005). Lack of recovery of species, such as Northern cod, has contributed to renewed interest in spatial stock structure in fisheries science (Cadrin, Kerr, & Mariani, 2014) as commercial fishing can erode local concentrations when spatial structure is ignored. Fishing patterns need to be evaluated with respect to stock structure so that smaller, more vulnerable concentrations and associated habitat are not overfished.

Halibut have been experiencing population growth, supported by a period of high recruitment in Canadian waters since the early 2000s (DFO, 2015a, 2015b; Trzcinski & Bowen, 2016). This upward trend is in contrast to the NW Atlantic continental shelf's documented ecosystem shift from one dominated by large‐bodied groundfish to one abundant in invertebrates (Frank, Petrie, Choi, & Leggett, 2005; Shackell & Frank, 2007; Shackell, Frank, Fisher, Petrie, & Leggett, 2010; Worm & Myers, 2003). Here, we present evidence of spatial structure in juvenile halibut in the NW Atlantic through three and a half decades of commercial exploitation by building on evidence that halibut are not habitat‐limited (Shackell et al., 2016) and that the majority of tagging studies suggest local residency. We also consider that the lack of halibut's recovery in the United States is likely due to historical local overfishing (Grasso, 2008).

Here, our goal was to explore the spatial structure of juvenile halibut abundance, an index of fisheries recruitment, using a hierarchical Bayesian spatiotemporal modeling approach (Carson & Mills Flemming, 2014; Cosandey‐Godin, Krainski, Worm, & Mills Flemming, 2015) to measure the persistence and connectivity of concentrations with high abundance. We used fishery‐independent groundfish research vessel trawl survey data of juvenile halibut from the United States and Canada to explore the spatial and temporal patterns of juvenile halibut abundance in the NW Atlantic over the last 36 years (1978–2013) across three different Canadian fisheries management regimes: (i) 1978–1989, post‐implementation of the EEZ; (ii) 1990–2003, low groundfish abundance; and (iii) 2004–2013, high halibut recruitment. With no a priori assumption about fisheries management units, including international borders, the model identified areas of relatively higher abundance that were persistent over time but the abundance of juvenile halibut varied among regimes. We argue that the protection of persistent high abundance areas may have contributed to the recovery of this stock and that sustainable management will need to consider stock structure.

## 2 METHODS

### 2.1 Data

Data from 27 fishery‐independent research trawl surveys conducted by the National Marine Fisheries Service (NMFS; USA) and the Department of Fisheries and Oceans Canada (DFO) were combined. The surveys are based on a stratified‐random sampling design, and sampling efforts were confined to within the continental shelf (Figure 1). The resulting time series employed a total of seven different trawl gear types which predominantly captured juvenile halibut (i.e., body size <80 cm). Surveys were conducted in different seasons and covered different years and geographic regions, namely Newfoundland and Labrador (NL), Nova Scotia (the Scotian Shelf and Gulf of Maine; NS), Northern Gulf of St. Lawrence (NGSL), Southern Gulf of St. Lawrence (SGSL), and the American and Canadian waters into the Gulf of Maine and Bay of Fundy sampled by NMFS. In total, 75,149 survey sets between 40N° and 52.25N° were sampled during the research survey time series from 1978 to 2013, 4,509 (6%) of sets caught juvenile Atlantic halibut (Table 1). Date, latitude, longitude, bottom temperature, depth, abundance (number), and biomass (weight in kg) were recorded for each set. Abundance was standardized within each research survey to account for variations in set duration and distance sampled (Ocean Biogeographic Information System (OBIS), 2014). Annual estimates of stratified mean abundances were used to display regional‐scale time series trends.

**Table 1:** Data Summary, region (ordered alphabetically), total sets, sets with halibut, percent with halibut, first year, last year in time series

Region	Total sets	Sets with halibut	Percent with halibut	Start year	End year
Newfoundland (NL)	34,170	1,142	3.34	1978	2013
Northern Gulf of St. Lawrence (NGSL)	3,487	385	11.04	1990	2013
Nova Scotia (NS)	12,965	2,126	16.40	1978	2013
Southern Gulf of St. Lawrence (SGSL)	6,802 (6,741)	494 (433)	7.26 (6.42)	1978	2013
United States of America (USA)	17,725	362	2.04	1978	2013
Summary	75,149 (75,088)	4,509 (4,448)	6.00 (5.92)		

The numbers in brackets represent the totals after removing 61 observations in the St. Lawrence River estuary (see Methods).

### 2.2 Approach: spatiotemporal model

To explore the abundance and distribution of juvenile Atlantic halibut, we employed a Bayesian hierarchical spatiotemporal model following the methodology of Carson and Mills Flemming (2014) (see also Cosandey‐Godin et al., 2015; Carson, Shackell, & Mills Flemming, in press). The model encompasses the 1978–2013 time series, which spans three time periods (or groups) defined by fisheries management regimes in Canada;

1. 1978–1989—The EEZ was established in 1977/1978. In the absence of foreign fleets, most groundfish stocks rebounded in this period, while Canada's domestic fleet capacity was building (Horsman & Shackell, 2009). Additionally, the halibut fishery was regulated in 1988, when management units and minimum size were defined by DFO. The research vessel surveys covered all areas except the NGSL, although in the SGSL, catches were very low (DFO, 2015b), *n* = 12 years, juvenile halibut abundance = 1,550 captures, and the number of surveys = 15.
2. 1990–2003—The first year for the NGSL trawl survey was 1990. Moratoria were declared on collapsed Newfoundland Atlantic cod fishery in 1992, on the Gulf of St. Lawrence (GSL) cod in 1993, and on groundfish on the Eastern Scotian Shelf in 1994, *n* = 14 years, juvenile halibut abundance = 1,298 captures, and the number of surveys = 16.
3. 2004–2013—This was a decade of high halibut recruitment and population recovery, *n* = 10 years, juvenile halibut abundance = 1,600 captures, and the number of surveys = 15.

**Figure 2:**
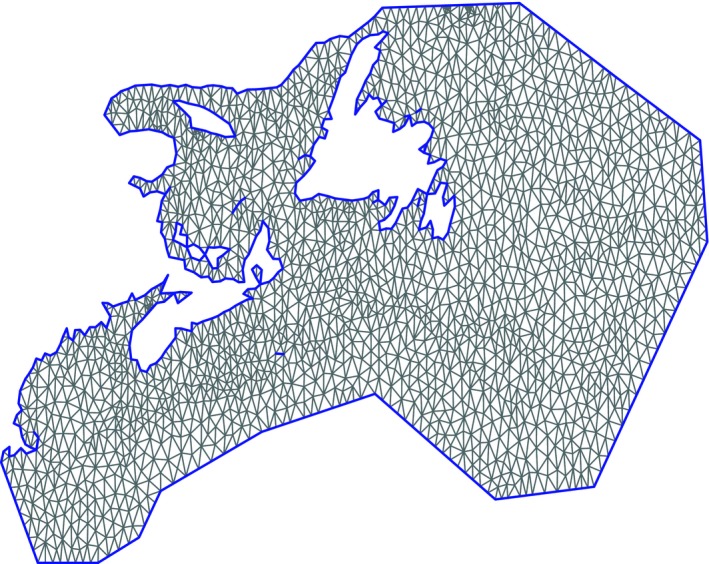
Triangulated mesh. Constrained refined Delaunay triangulation mesh. The smaller triangles of the grid are where there are more data points. It is formed of triangles, and the vertices are called nodes (here there are 2,120)

We used an Integrated Nested Laplace Approximation (INLA; Rue, Martino, & Chopin, 2009) approach available in the R Statistical Programming software's R‐INLA package (Martins, Simpson, Lindgren, & Rue, 2012; R Core Team 2013). R‐INLA has been used recently in the marine environment to model fisheries bycatch, population abundance, and animal movement (e.g., Carson & Mills Flemming, 2014; Quiroz, Prates, & Rue, 2014; Cosandey‐Godin et al., 2015). R‐INLA uses a stochastic partial differential equations (SPDE) approach to model spatial dependence on a triangulated mesh. The mesh is built from the data of the model domain (Figure 2), and ultimately facilitates the production of maps to visualize the underlying spatiotemporal structure. To avoid high variance near the boundary, or boundary effects, the model domain was extended beyond the locations of data on the continental shelf, and the sharp corner of the St. Lawrence River estuary was omitted (see Lindgren & Rue, 2015). Exclusion of the data in the St. Lawrence River estuary removed 61 observations (visible in Figure 1) resulting in an analysis of 4,448 observations (Table 1).

For spatial analyses of large datasets, SPDE models are efficiently fit using a Gaussian random field (GRF), a discretely indexed spatial process. The GRF models spatial dependence on a mesh and estimates the parameters of the covariance function (i.e., the Matérn covariance function; Rue & Held, 2005; see also Lindgren, Rue, & Lindstrom, 2011 for theory and proofs; and www.r‐inla.org). Following Carson and Mills Flemming (2014; Carson et al., in press), we estimated connectivity (coherence of temporal pattern over space) and spatial variance (variation across the seascape) of juvenile halibut abundance across the modeled domain. In addition, a temporal autocorrelation term in the model allowed us to estimate the persistence (similarity of spatial structure over time) of the areas of abundance.

The response variable of interest is juvenile halibut abundance (number) at each location in each time period. All true zero values in the original data were removed from the dataset, but were represented in the spatiotemporal model as nonpositives in the mesh (see Figure 2, smaller triangles where data are positive). As nonpositive values are computationally less demanding, this enabled us to run the analysis.

The model has the following general form;

$$E\left(Y_{s,t}\right)=\xi \left(s,t\right)+\sum_{j=1}^{n}f_{i}\left\{c_{j}\left(s,t\right)\right\},$$

where *E*(*Y*_*s*,*t*_) is the mean of the expected response of *Y* at location *s* at time *t*, ξ(*s*,*t*) represents the spatiotemporal latent GRF (a random effect), *f*_*j*_ (*c*_*j*_(*s*,*t*)) are smoothed functions of the covariates, while *j* refers to *j*th of a total *n* covariates where depth and temperature were the tested covariates. The mean of the response variable, *E*(*Y*(*s*,*t*)), is mapped by a canonical link function (i.e., log link) to a linear predictor, η(*s*,*t*), by the generalized additive model framework (Hastie & Tibshirani, 1990). The spatiotemporal latent GRF, ξ(*s*,*t*), represents the cumulative effect of all unmeasured latent factors. The characteristics of this spatiotemporal random effect comprise the spatial and temporal covariance structure of the model (Rue et al., 2009).

In addition to defining the domain and building the mesh, it was also necessary to determine the appropriate statistical distribution of the response variable, the importance of covariates, and the inclusion of the latent field to the model. The error distributions explored included Poisson, negative binomial, and Gaussian. The spatiotemporal covariance structure was also tested, examining whether it was temporally invariant (a single spatial field), and whether the time periods were autocorrelated or independent (see Cosandey‐Godin et al., 2015).

#### 2.2.1 Persistence

Through this process of model building and evaluation, the autocorrelation of the abundance and location of halibut through the three time periods (*t* = 3) was tested to determine whether the locations persisted from one time period to the next, as measured by the AR(1) term. The AR(1) term, a first‐order autoregressive model using the previous time step to predict the most recent, is interpreted as the *a* parameter, or persistence, which ranges from −1 to 1.

#### 2.2.2 Goodness of fit

The goodness of fit of the various candidate models was compared using Bayesian diagnostics, namely the deviance information criterion (DIC) and conditional predictive ordinate (CPO; the logarithm of the pseudomarginal likelihood; see Carson & Mills Flemming, 2014; Carson et al., in press). The lower DIC and larger CPO indicate a better the model fit. Both DIC and CPO are calculated by R‐INLA.

#### 2.2.3 Connectivity and spatial variance

R‐INLA allows estimation of spatial characteristics. Connectivity, or ρ, is conventionally interpreted as the distance at which the spatial covariance of the field decays to 0.13 (Cameletti, Ignaccolo, & Bande, 2011; Cameletti, Lindgren, Simpson, & Rue, 2013). When the connectivity parameter, ρ, is large, it means that the covariance decays slowly in space, that is, the temporal pattern is similar over a large spatial scale. The units of connectivity are degrees Latitude, which are converted to distance in kilometers (km) for added interpretation. Similarly, σ^2^, spatial variance is a relative index of the differences in amplitude across a seascape, and is scaled to the linear scale of the predictors (i.e., log juvenile halibut abundance). A large spatial variance indicates a large amplitude in the overall field. When ρ and σ^2^ are reported as parameters of the model, they provide a description of the multivariate normal distribution of the mean of the response, after accounting for the variables which are explicitly included. Our model, incorporating three fisheries management regimes, has one ρ (index of connectivity) and σ^2^ (spatial variance), in addition to one *a* (persistence) parameter. Bayesian credibility intervals indicating the probability that the parameters lie within a specific range were also examined. To examine the connectivity, spatial variance, and persistence of juvenile Atlantic halibut areas of abundance through the three time periods, these random latent fields were plotted as maps.

## 3 RESULTS

### 3.1 Time series of abundance indices

**Figure 3:**
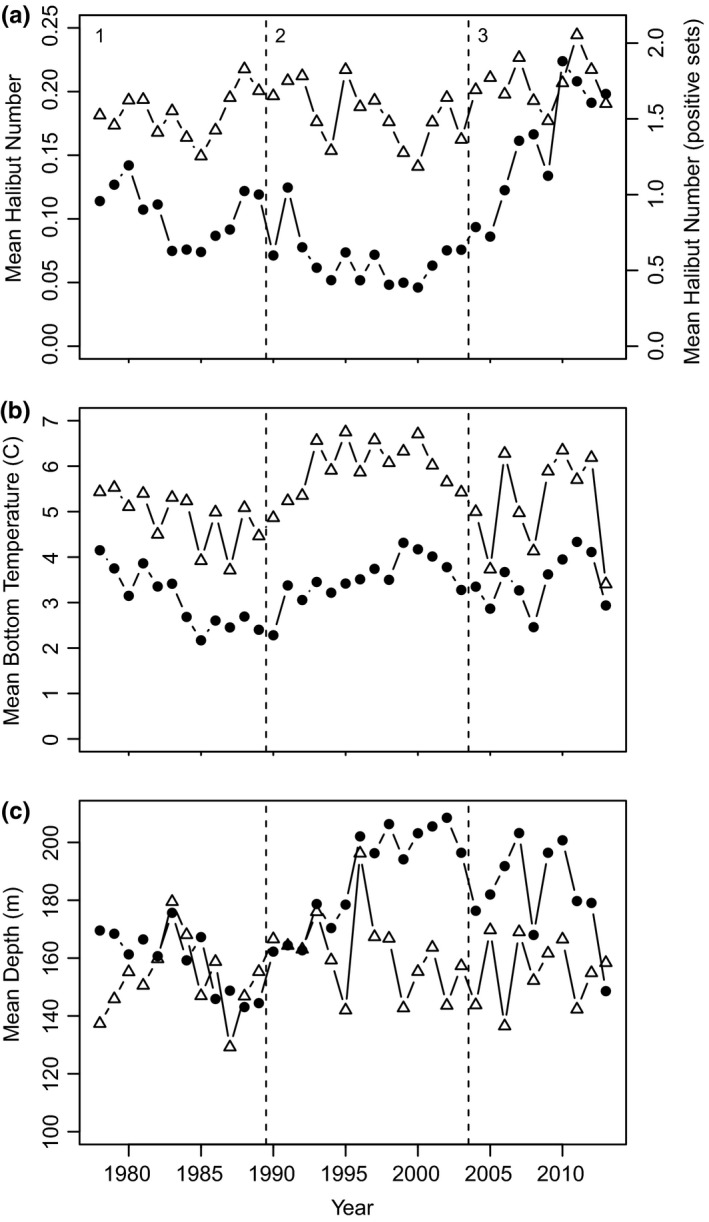
Time series trends. Stratified mean trends in juvenile Atlantic halibut from the trawl surveys by year using all data, presence ≥0 (closed circles), and positive sets only (open triangles). (a) The stratified mean number of halibut from all sets on the left *y*‐axis, and calculated for the positive sets only on the right‐hand *y*‐axis, (b) stratified mean bottom temperature (°C), and (c) stratified mean depth (m). Time periods are: (i) 1978–1989, (ii) 1990–2003, and (iii) 2004–2013

Halibut were captured most often along the continental shelf, associated with a deeper edge, and also within the GSL (Figure 1). Stratified means of halibut abundance decreased during 1989–2003 relative to the first time period, and have since been increasing (Figure 3a). Halibut generally have a low catchability in trawl surveys, with the stratified mean abundance ranging from 0.05 to 0.25 halibut captured. Of the positive sets, the mean ranged from 1.19 to 2.05 halibut (Figure 3a). Stratified mean bottom temperature of all stations (zero and nonzero) was generally higher and more consistent during the second time period; however, temperatures throughout the study have remained between 2 and 5°C (Figure 3b). In the stations with halibut present, mean temperatures were warmer than those on average in all of trawl surveys, ranging between 3.41 and 6.75°C (Figure 3b). The stratified mean depths sampled in all of the surveys ranged between 140 and 210 m (Figure 3c), with the deeper sampled sets most consistently taking place during the second period (1990–2003). In the positive sets, halibut were at mean depths of 129.2 to 196.2 m (Figure 3c). Surveys in Nova Scotian waters (the Scotian Shelf and Gulf of Maine) had the highest percent occupied, 16%, while the United States had the lowest, 2% (Table 1).

### 3.2 Model selection

First models were tested using alternative likelihood families, temporal structures, covariates, and inclusion of the latent effect. The best performing models had a spatiotemporal random field using a first‐order autoregression model that yielded, *a*, the persistence parameter (Tables 2 and 3). The latent field ξ(*s*,*t*) of each time period (group) was a function of the value of the one previous (i.e., correlated). The DICs and CPOs of models using the Poisson error distribution were consistently better than those for other error distributions. The covariates were largely insignificant most likely because the model focussed on nonzero values. As per the resulting lowest DIC and highest CPO, bottom temperature and the latent variable were retained in the penultimate model (Table 2); η(*s*,*t*) = ξ(*s*,*t*) + Bottom Temperature. A smoothing function was not applied to bottom temperature.

**Table 2:** Covariates, DIC and CPO values for various candidate models with an AR(1) spatiotemporal covariance structure and a Poisson likelihood

Covariate(s)	DIC	CPO
ξ(*s*,*t*) + temperature	**13433.31**	**−6735.08**
ξ(*s*,*t*) + depth + temperature	13435.71	**−6735.08**
ξ(*s*,*t*)	13446.01	−6741.99
ξ(*s*,*t*) + depth	13447.82	−6742.17

Models are listed in order of lowest to highest DIC. Bolded values indicate the lowest DIC and the highest CPO and are the better fitting models.

**Table 3:** Model posterior estimates (mean, standard deviation, and 95% credible interval) of the penultimate model

Model parameters	Mean	*SD*	*Q*_0.025_	*Q*_0.5_	*Q*_0.975_
ρ Connectivity	2.25 (249.75 km)	0.44	1.55	2.19	3.26
σ^2^ Spatial variance	0.12	0.02	0.09	0.12	0.16
*a* Persistence	0.77	0.06	0.64	0.78	0.88

The model has an AR(1) spatiotemporal structure, Poisson likelihood, and includes the latent field, and bottom temperature.

**Figure 4:**
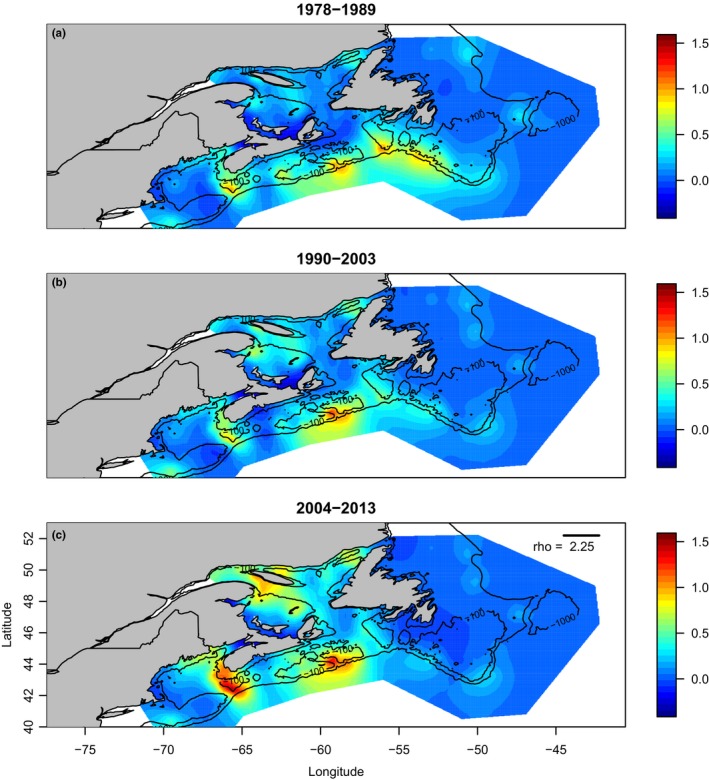
Random fields of juvenile halibut abundance of the penultimate model by fisheries management time period, the model parameter connectivity, ρ (rho), is drawn and printed on the bottom map, units are degrees Latitude. The color scale illustrates the random latent field (log abundance), and all three panels are the same scale. The log abundance for panel (a) time period (1) 1978–1989 ranged from −0.30 to 1.07 with a mean of 0.16, for (b) (2) 1990 to 2003; ranged from −0.37 to 1.27 with a mean of 0.15, and (c) (3) 2004–2013; ranged from −0.25 to 1.55 with a mean of 0.21. Bathymetric contours illustrate the 100 and 1,000 m depths representing the continental shelf and larger banks

Visual analysis of the areas of abundance through the three periods illustrates that juvenile halibut distributions have changed over time (Figure 4). Examining the model results, connectivity (ρ) showed that, on average, nodes in the random field domain were significantly spatially correlated up to 250 km (2.25° Latitude) (Table 3; Figure 4). The spatial variance (σ^2^) was also quite low (0.12) indicating a relatively flat field (Table 3). Persistence (*a*) was relatively high (0.77) with a small standard deviation (0.06), indicating that juvenile halibut were present in about the same locations in each time period and were correlated through time (Table 3). In the first time period, 1978–1989 (Figure 4a), there appear to be five locations with a high abundance on the Scotian Shelf and in southeastern Newfoundland following the continental shelf edge, while there was very low abundance in the GSL (Figure 4a). During 1990–2003 (Figure 4b), patches of halibut abundance are identified in the GSL and United States. The area of higher abundance was reduced to two core areas on the Scotian Shelf, the Gully and off SWNS. Finally, in the most recent period (2004–2013; Figure 4c), juvenile halibut abundance increased, and again, two high abundance areas were identified in SWNS and the Gully. While halibut abundance has increased in GSL through time, it has remained low off NL.

### 3.3 Spatiotemporal model results

The analysis over three time periods is an initial broad look at the spatiotemporal trends in juvenile halibut abundance in the NW Atlantic. Two areas of persistent abundance were identified, both on Scotian Shelf: (i) Southwest Nova Scotia and (ii) the Gully (see Figures 1 and 4). Less persistent high abundance areas were also found in the GSL and NL, although the results may be confounded by differences in surveys. In Newfoundland, there are areas of abundance in 1978–1989 (time period 1) which do not persist (Figure 4). While in the GSL, patches of high abundance have appeared more recently (2004–2013) in both the NGSL and SGL (Figure 4).

## 4 DISCUSSION

Here, we demonstrate that halibut exhibit a spatial structure in the NW Atlantic at a scale smaller than the current halibut stock management units. Our analysis showing statistically independent persistent areas of juvenile halibut abundance has provided important evidence that halibut spatial structure is more complex than previously identified, and has varied over the past three and a half decades. Two high abundance areas were consistently present on the Scotian Shelf throughout the time series, southwest Nova Scotia and the Gully (Figure 4). Despite widespread increase in halibut recruitment during the last decade, previously identified areas of high juvenile abundance have not re‐established in southern Newfoundland (Figure 4c). Connectivity of juvenile halibut is an order of magnitude less than the distance assumed by the definition of the management units (~2,000 km; DFO, 2015a). Halibut have a long history of being removed as bycatch in the cod‐directed groundfisheries (Grasso, 2008) and persistent patches of abundance along the shelf edge may identify spatial refuges from cod‐directed fishing. Coinciding with the reduction in trawling and cod fisheries in the early 1990s, and the introduction of a minimum legal size for landed halibut, halibut have increased since the mid‐2000s and catches in the Canadian trawl surveys have been well above the long‐term average (DFO, 2015a, 2015b; see also Figures 3 and 4).

While a substantial amount of knowledge is required to identify a discrete population unit, stock assessments, sustainable seafood classifications (i.e., Marine Stewardship Council), and vulnerable species classifications depend on knowing the demographic rates for identified stocks or subpopulations and the impact of fisheries on them. Sustainable management and recovery of fish stocks are further complicated when fine‐scale differences in local populations are observed such as in the Skagerrak cod (Olsen et al., 2008) and yellowfin sole (*Limanda aspera*) in the Bering Sea (Bartolino, Ciannelli, Spencer, Wilderbuer, & Chan, 2012). In the Northeast Atlantic, evidence of genetic differentiation provides support for the existence of local populations of Atlantic halibut (Foss, Imsland, & Nævdal, 1998; Haug & Fevolden, 1986; Mork & Haug, 1983). In the NW Atlantic, Reid et al. (2005) found no genetic evidence supporting local populations but acknowledged that in the absence of spawning information and with limited sample size, the power to detect differences was not ideal. Genetically distinguishable spawning populations that mix after spawning may not easily be assigned to a home location (Reid et al., 2005). More research is required to determine the genetic population structure of Atlantic halibut.

Even in the absence of genetic differentiation, high abundance areas are especially vulnerable to overfishing if connectivity between areas is low and fishing pressure is high (Shackell et al., 2005). Rapid population‐level declines have been observed in demersal fish on the Scotian Shelf (Reuchlin‐Hugenholtz, Shackell, & Hutchings, 2015) and cod in Newfoundland (Hutchings, 1996). Notably, one of the persistent high abundance areas is adjacent to US waters, where lack of recovery in halibut since the 1800s is evidence for local overfishing (Shackell et al., 2016; Seitz et al., 2016).

Tagging data provides evidence of connectivity between Canada and United States with almost 30% of the recaptures of halibut tagged in New England occurring in Canadian waters (Kanwit, 2007). However, in general, tagging studies suggest that a high proportion of halibut are resident or return seasonally to particular locations, with the majority of halibut captured within 200 km of where they were released, and a small proportion moving large distances across the management unit and beyond (den Heyer et al., 2012; Stobo, Neilson, & Simpson, 1988). In the GSL during 2013–2015, a study employing satellite tags recorded pop‐off locations which were <55 to 423 km from the site of tagging, and local residency was inferred (Murphy et al., 2017). Of note, discrete spawning units of Pacific halibut have been suggested through satellite tagging, supported, in part, by low rates of spawning dispersal across the geographic regions (Seitz et al., 2017).

The statistical independence of points on the random field outside of ~250 km in the present analysis infers a more complex population structure but does not explicitly incorporate the two distinct stock management paradigms of the GSL and Scotian Shelf‐southern Grand Banks. The analysis does however capture the changing abundance in both management regions (i.e., DFO, 2015a, 2015b; Trzcinski et al. 2011, see also Figure 4). During the 1980s and 1990s, the NW Atlantic ecosystem shifted from large‐bodied predators to crustaceans, largely due to overexploitation (Frank et al., 2005). The observed decline in groundfish abundance (Frank et al., 2005; Shackell & Frank, 2007) and body size (Shackell et al., 2010) was followed by a large increase in benthic decapods and other prey species, likely because of predation release (Boudreau & Worm, 2010; Steneck, Vavrinec, & Leland, 2004; Worm & Myers, 2003). In recent years in the NW Atlantic, there have been some signs of groundfish population growth, namely in haddock (*Melanogrammus aeglefinus*; DFO, 2012), cod (Cadigan, 2016; Rose & Rowe, 2015), and halibut (DFO, 2015a, 2015b; Trzcinski & Bowen, 2016).

We propose that the persistent areas of high juvenile abundance on the Scotian Shelf were offered protection from commercial fishing, and trawl gear, and that this protection has contributed to the rebounding of this stock. One persistent area overlaps with a groundfish seasonal spawning closure, while the other occurs in the Gully, a deep water canyon and marine protected area adjacent to a closed area to protect juvenile haddock (O'Boyle, 2011). We note however that the Gully does permit some longline fishing in the outer zones (Department of Fisheries and Oceans Canada (DFO), 2008), but the fishery is prosecuted with a minimum size limit of 81 cm. Notably, the Gully persistent area occurs on the eastern Scotian Shelf, where commercial groundfish fisheries have been drastically reduced (O'Boyle, 2011), and this could contribute additional protection of juvenile halibut from capture as bycatch in other fisheries. Additionally, halibut, unlike cod (Hutchings, 1996), do not spawn in groups (Trumble et al., 1993) which has likely also served as a refuge from fishing pressure.

Persistent communities of juvenile halibut have remained in SWNS and the Gully (Figures 3 and 4), suggesting that these areas are core high abundance refugia and density‐dependent habitat selection is occurring. As these preferred high abundance areas becomes increasingly occupied, resulting in limited resources, halibut may begin to occupy less ideal habitat in order to reduce intraspecific competition (Fisher & Frank, 2004; Gaston, 2003). Density‐dependent habitat selection is generally assumed to be associated with preferred habitats that are rich in prey. Juvenile halibut up to 30 cm in length feed almost exclusively on invertebrates, those 30 to 80 cm in length feed on both invertebrates and fish, while halibut larger than 80 cm in length feed almost exclusively on fish (Kohler, 1967). It has yet to be examined how juvenile halibut abundance and distribution have covaried with their preferred prey species as the NW Atlantic's ecosystem shifted. Building on the analyses presented here, a closer examination of regions with persistent high juvenile abundance or historically high halibut abundance, such as southeastern Newfoundland (Figure 4), could find evidence of local overfishing, differing demographic rates between areas of high abundance, or other finer scale spatiotemporal dynamics of prey.

## 5 CONCLUSION

Using a statistically powerful tool and more than three decades of standardized groundfish trawl survey data from Cape Cod to the Grand Banks, we identified areas of high juvenile halibut abundance that have persisted through three fisheries management regimes. Other high‐density areas were diminished and, as of yet, have not re‐established. As more research is needed to define subpopulations, and to prevent serial local overfishing in the future, we propose a pragmatic interim approach; one that focuses on protecting important patches where density of fish is persistently high (i.e., high abundance areas), indicating preferred or ideal resources such as prey and a refuge from predators. These areas could also serve to be refuges from fishing pressure, both naturally, such as a deep water canyon (i.e., the Gully), or intentionally as part of a fisheries management strategy to protect juveniles. Atlantic halibut in Canadian waters are experiencing a period of high recruitment and population growth, a relatively unique and positive trend for a commercial fish in the depleted NW Atlantic ecosystem, and there is an opportunity to manage them in a precautionary way. The history of serial depletion of Atlantic halibut underscores the need to consider stock structure in its management. Given the ecological consequences of serial overfishing, local concentrations of halibut should be safe‐guarded until it is known whether the evident spatial structure represents distinct or connected populations. We argue that areas of persistently high abundance of juveniles should receive some protection in order to sustain the recovered population(s) today and in the future.

## CONFLICT OF INTEREST

None declared.

## AUTHOR CONTRIBUTIONS

SAB, NLS, and CEdH conceived the research project; SAB and SC performed the analyses. All authors contributed to the interpretations of the results and assisted SAB in composition of the manuscript.
